# Molecular mechanisms of folliculogenesis and oogenesis

**DOI:** 10.1080/19396368.2026.2631558

**Published:** 2026-04-02

**Authors:** Mohamed Aboul Ezz, Ahmed Zaky Balboula

**Affiliations:** a Division of Animal Sciences, University of Missouri, Columbia, Missouri, USA;; b Department of Theriogenology, Faculty of Veterinary Medicine, Mansoura University, Mansoura, Egypt

**Keywords:** Follicle, oocyte, cumulus cells, meiosis, spindle, developmental competence

## Abstract

Folliculogenesis is a complex, multi-stage process crucial for the establishment and maintenance of female fertility through the production of a developmentally competent oocyte. Folliculogenesis, including follicular formation, activation, growth and maturation, relies on a finely tuned spatiotemporal crosstalk between germ cells, somatic cells, and the hypothalamic-pituitary-ovarian axis. This work provides a comprehensive overview of the cellular dynamics and molecular mechanisms underlying each stage of follicular development. A particular emphasis is placed on the interaction of growth factors, transcriptional networks, signaling pathways and endocrine cues that collectively govern follicular fate and oocyte quality. Disruptions in these interactions lead to emergence of pathological conditions such as premature ovarian insufficiency and age-related infertility. We further highlight the dual aspects of oocyte maturation, nuclear and cytoplasmic, as major determinants of developmental competence, and explore the role of spindle dynamics, organelle redistribution and epigenetic reprograming in this process. The bidirectional communication between oocytes and cumulus cells, mediated by paracrine signaling and jap junctions, is underscored as a pivotal regulator of oocyte metabolic activity, redox homeostasis, and meiotic competence. A better understanding of the oocyte-cumulus cell interaction offers new approaches for refining the *in vitro* maturation systems and improving assisted reproductive technologies. A special attention is given to the emerging use of cumulus cell-derived biomarkers for noninvasive assessment of oocyte quality and prediction of preimplantation embryo development. Taken together, this article presents an integrated framework to guide future research in reproductive biology, regenerative medicine, and fertility preservation.

## Introduction

The ovary serves for two main functions: (1) producing female gametes (eggs), and (2) synthesis and secretion of sex hormones. Both of these vital functions are governed by the ovary’s functional units: the ovarian follicle and the corpus luteum (Edson et al. 2009; Richards and Pangas 2010). Given that corpus luteum formation requires an ovulated follicle, folliculogenesis, the process by which ovarian follicles develop, is a cornerstone of female fertility (McGee and Hsueh 2000).

Folliculogenesis is a highly dynamic process involving effective coordination between the germ cells, somatic cells, and the hypothalamic-pituitary-ovarian axis (Rajkovic et al. 2006). This coordination must be tightly and timely regulated (Sánchez and Smitz 2012). Disruption of these processes can lead to pathological conditions like premature ovarian insufficiency and age-related infertility (Pangas and Rajkovic 2006).

This chapter will go through the fundamental biology of folliculogenesis and oogenesis, shedding light on the cellular events and molecular mechanisms that orchestrate the development of female gametes within the follicle. Special attention will be paid to the bi-phases of oocyte maturation, both nuclear- and cytoplasmic maturation, which are necessary for gaining the developmental competence (Zuccotti et al. 1998; Sánchez and Smitz 2012; Conti and Franciosi 2018). Central to this discussion is how interactive communication between oocytes and their surrounding cumulus cells is established, and the impact of this dialogue on follicular growth and oocyte quality.

## Biology of folliculogenesis

### Formation of primordial follicles

Primordial germ cells (PGCs), the precursors of female gametes (oocytes), are specified from the epiblast as early as 7.5 days of embryonic life (E7.5) in mice. This specification is triggered by signals from adjacent tissues, particularly bone morphogenetic proteins (BMPs). BMP4, BMP8b (derived from extra-embryonic ectoderm), and BMP2 (derived from visceral endoderm) are required for PGC induction (Lawson and Hage 1994; Ying et al. 2000; Ying and Zhao 2001; Kobayashi et al. 2017). Around E10.5, PGCs migrate to the genital ridge of undifferentiated gonads where they undergo mitosis to form oogonia. Concurrently, in the absence of Y-linked gene ‘Sry’ which is required for development of male gonads (testes), the XX genital ridges differentiate into female gonads (ovaries) under the influence of somatic cell derived transcription factors (detailed in Table 1) (Elvin and Matzuk 1998; Achermann and Jameson 1999; Bowles and Koopman 2010). After PGCs colonization at the embryonic ovary, female germ cells undergo mitotic divisions with incomplete cytokinesis forming clusters or syncytia, called ‘germ cell cysts’ or ‘germ cell nests’ (Pepling 2006). Within the nests, the oocytes remain connected by intercellular bridges, facilitating the transfer of cytoplasmic organelles (Pepling and Spradling 1998; Epifano and Dean 2002; Lei and Spradling 2016).

Around E13.5, oocytes within germ cell nests enter the prophase of meiosis I, which includes leptotene, zygotene, pachytene, diplotene, and diakinesis stages. By E18.5, oocytes proceed to the diplotene stage, then enter a prolonged resting phase called dictyate (Hunt and Hassold 2008). Oocytes remain arrested at prophase stage until preovulatory LH surge induces meiotic resumption (Jagarlamudi and Rajkovic 2012). Simultaneously, germ cell nests start to disintegrate forming the primordial follicles in which oocytes are surrounded by a single layer of squamous pre-granulosa cells derived from the ovarian surface epithelium (Niu and Spradling 2020). In mice, this process is completed predominantly during the first few days after birth, establishing the primordial follicle pool (Pepling 2006).

### Activation of primordial follicles

Following establishment of the primordial follicle pool, each follicle may follow one of these four pathways: (1) remain dormant for variable lengths of time as part of the ovarian reserve, (2) experience immediate atresia, (3) be recruited for growth and eventually ovulate, or (4) undergo initial recruitment followed by atresia. Remarkably, less than 1% of ovarian follicles attain ovulation, whereas over 99% are lost due to atresia (Yu et al. 2004; Zhou et al. 2019; Harris et al. 2023).

The earliest sign of primordial follicle activation during initial recruitment is the morphological change of granulosa cells from squamous to cuboidal epithelium. Concurrently, the oocyte enlarges and acquires a zona pellucida, a thick glycoprotein layer necessary for fertilization accomplishment. The initial recruitment is achieved when the follicle encompasses an oocyte surrounded by a single layer of cuboidal granulosa cells, known as primary follicle (Hirshfield 1991; Rajah et al. 1992; Fortune 1994, 2003). Primordial to primary follicle transition is the longest phase in folliculogenesis, taking over six months in humans and nearly two weeks in mice (Williams and Erickson 2000).

### Primary to secondary follicle transition

The rapid proliferation of granulosa cells allows the transition from primary to secondary follicles. The secondary follicle, consisting of growing oocyte covered by multiple layers of granulosa cells. Later, the basement membrane is established to separate between the granulosa cells (inside the membrane) and a newly formed somatic cell layer, theca cells (outside the membrane). The theca cell layer is classified into theca interna (facing the membrane) and theca externa (facing the ovarian stroma). While theca cell layer is highly vascularized, blood vessels do not penetrate beyond the basement membrane (Kol and Adashi 1995; Magoffin 2005; Onagbesan et al. 2009; Knight et al. 2012).

### Antral development and preovulatory follicle formation

Antral follicles are characterized by the fusion of fluid-filled cavities, appearing in early antral follicles, to form the antrum as well as granulosa cell differentiation into two subcategories: cumulus cells surrounding the oocyte (cumulus granulosa cells) and mural granulosa cells lining the follicular wall (Rimon-Dahari et al. 2016). Like the earlier follicular stage, most antral follicles experience atresia with only a small percentage develops to mature Graafian follicles, which eventually ovulate (Richards 1980; Zeleznik 2001). Ovulation process encounters a series of events, including oocyte maturation, cumulus cell expansion, follicle rupture, and oocyte extrusion (Robker et al. 2018; Duffy et al. 2019).

**Figure 1.**
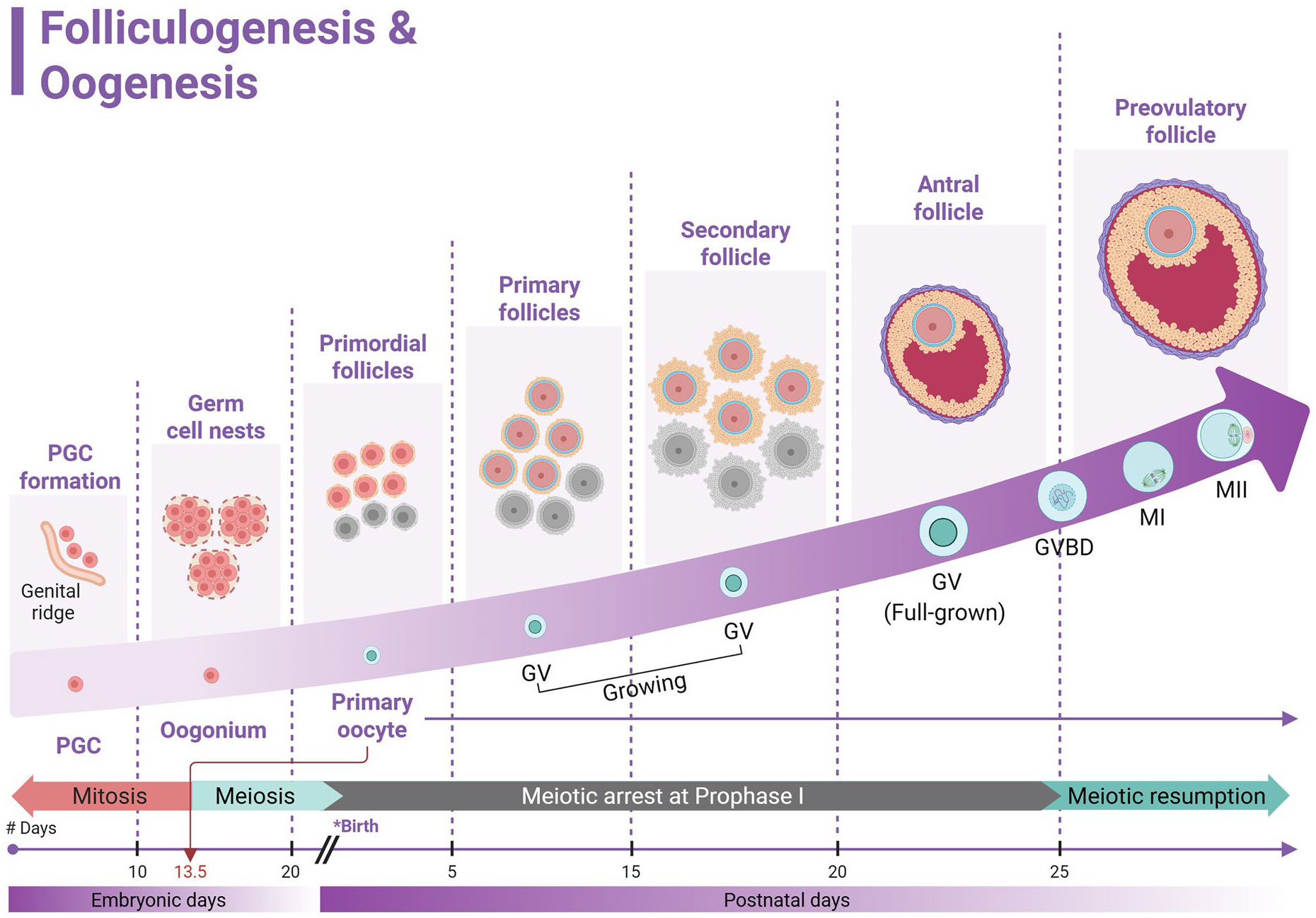
A schematic model for folliculogenesis and oogenesis in mice. At 7.5 days of embryonic life (E7.5), the extra-embryonic primordial germ cells (PGCs) migrate to the genital ridge. By E10.5, PGCs colonize the gonads and proliferate *via* mitosis to form clusters of oogonia, called germ cell nests. Around E13.5, the oogonia initiate meiosis and differentiate into oocytes. After birth, oocytes arrest in the dictyate stage of meiotic prophase I, also known as germinal vesicle (GV) stage, and remain arrested until puberty. Simultaneously, the germ cell nests disintegrate establishing a definite pool of primordial follicles, a key step in folliculogenesis process. Upon follicular activation, the primordial follicles grow and mature though primary, secondary, and antral stages, eventually forming preovulatory follicles. Throughout folliculogenesis, over 99% of ovarian follicles undergo atresia (marked in gray), while less than 1% progress to ovulation. During the ovulatory cascade, surge luteinizing hormone stimulates resumption of meiosis. The oocytes undergo germinal vesicle breakdown (GVBD), progress to metaphase I (MI), and arrest again at metaphase II (MII) until fertilization.

It has been reported that in humans, primordial follicles are detectable by approximately 20 weeks of fetal life, while primary follicles can be identified by around 24 weeks of gestation, with a subset progressing to the secondary stage by approximately 26 weeks. Antral follicles emerge during the third trimester of pregnancy and may undergo limited postnatal development during early infancy, coinciding with the physiological rise in follicle-stimulating hormone (FSH). However, sustained antral growth and selection of preovulatory follicles occur only after puberty, driven by cyclic gonadotropin stimulation during the menstrual cycle (McGee and Hsueh 2000). Notably, there is considerable discrepancy in the reported timing and prevalence of early human follicular stages across studies, likely reflecting differences in sampling, histological criteria, gestational age estimation, and analytical approaches (Kurilo 1981; Konishi et al. 1986; Abir et al. 2002; Fulton et al. 2005). The sequential morphological and cellular events of folliculogenesis from primordial follicle formation through ovulation, as modeled in the mouse ovary, are summarized in Figure 1.

## Regulation of folliculogenesis process

### Growth factors

Members of the transforming growth factor-beta (TGF-β) superfamily including BMP2, BMP4, BMP7, and BMP15 as well as growth differentiation factors 9 (GDF9) play essential roles in early folliculogenesis (i.e., from primordial till preantral follicular development). Lack or downregulation of these genes impairs follicular development (Otsuka et al. 2000; Yan et al. 2001; Pesce et al. 2002; Orisaka et al. 2006; Ross et al. 2007; Rossetti et al. 2009). On the other hand, insulin-like growth factors (IGF1 and IGF2) (Zhou et al. 1997; Kwintkiewicz and Giudice 2009) and vascular endothelial growth factor (VEGF) (Geva and Jaffe 2000) are crucial for the formation of antral and preovulatory follicles.

### Transcription factors

Alongside growth factors, a wide range of transcription factors originating from germ- and/or somatic cells orchestrates the folliculogenesis process in mammals (Table 1).

### Key signaling pathways

#### PI3K signaling

The phosphatidyl-inositol 3-kinase (PI3K) signaling pathway controls the balance between follicular dormancy and activation. Forkhead box O3 (FOXO3), a key molecule in this pathway, keeps primordial follicles in a dormant state. When the follicle is ready to grow, PI3K stimulates the conversion of phosphatidylinositol (4,5)-bisphosphate (PIB2) into phosphatidylinositol (3,4,5)-trisphosphate (PIB3) (Castrillon et al. 2003). PIB3 activates protein kinase B (AKT), which in turn in phosphorylates FOXO3; such phosphorylation translocases FOXO3 from the nucleus to cytoplasm where it becomes inactive, allowing the follicle to develop (Robertson 2005). Phosphatase and tensin homolog deleted on chromosome 10 (PTEN), a negative regulator of PI3K, prevents premature activation by inhibiting PI3K-AKT interaction (Robertson 2005). *FOXO3* or *PTEN* knockout leads to premature ovarian insufficiency (POI) *via* depleting the follicular reserve in young adulthood. On the contrary, *FOXO3* or *PTEN* overexpression impairs the follicular growth and development (Reddy et al. 2008; Pelosi et al. 2013).

#### mTORC1 signaling

The mammalian target of rapamycin complex 1 (mTORC1) drives follicular growth and maturation, while tuberous sclerosis complex (TSC1 and TSC2) suppresses mTORC1 pathway activation, thereby maintaining the follicular quiescence (Crino et al. 2006; Adhikari et al. 2010; Laplante and Sabatini 2012). In a similar way to FOXO3 and PTEN, deletion of *TSC1* and *TSC2* in mouse ovaries causes premature activation of primordial follicles due to excessive stimulation of mTORC1, which can potentially shrink the ovarian reserve over time (Adhikari et al. 2009; Gorre et al. 2014).

#### KIT/KITL signaling

The receptor tyrosine kinase (KIT), expressed in both germ- and somatic cells, plays a crucial role in follicular development upon binding to its ligand, KITL (also known as stem cell factor; SCF) (Matsui et al. 1990; Buehr et al. 1993). KIT/KITL pathway mediates bidirectional communication between germ cells and surrounding somatic cells. KITL, produced by granulosa cells, binds to KIT receptors on the oocyte membrane, thereby stimulating follicular development. Disruption of this pathway, as seen in *KIT/KITL* mutant mice, leads to arrested follicular development at the primary follicle stage. Conversely, exogenous KITL treatment in neonatal ovaries increases the number of growing follicles. KITL can also act on stromal cells, promoting their differentiation into theca cells (Nilsson and Skinner 2004).

**Figure 2.**
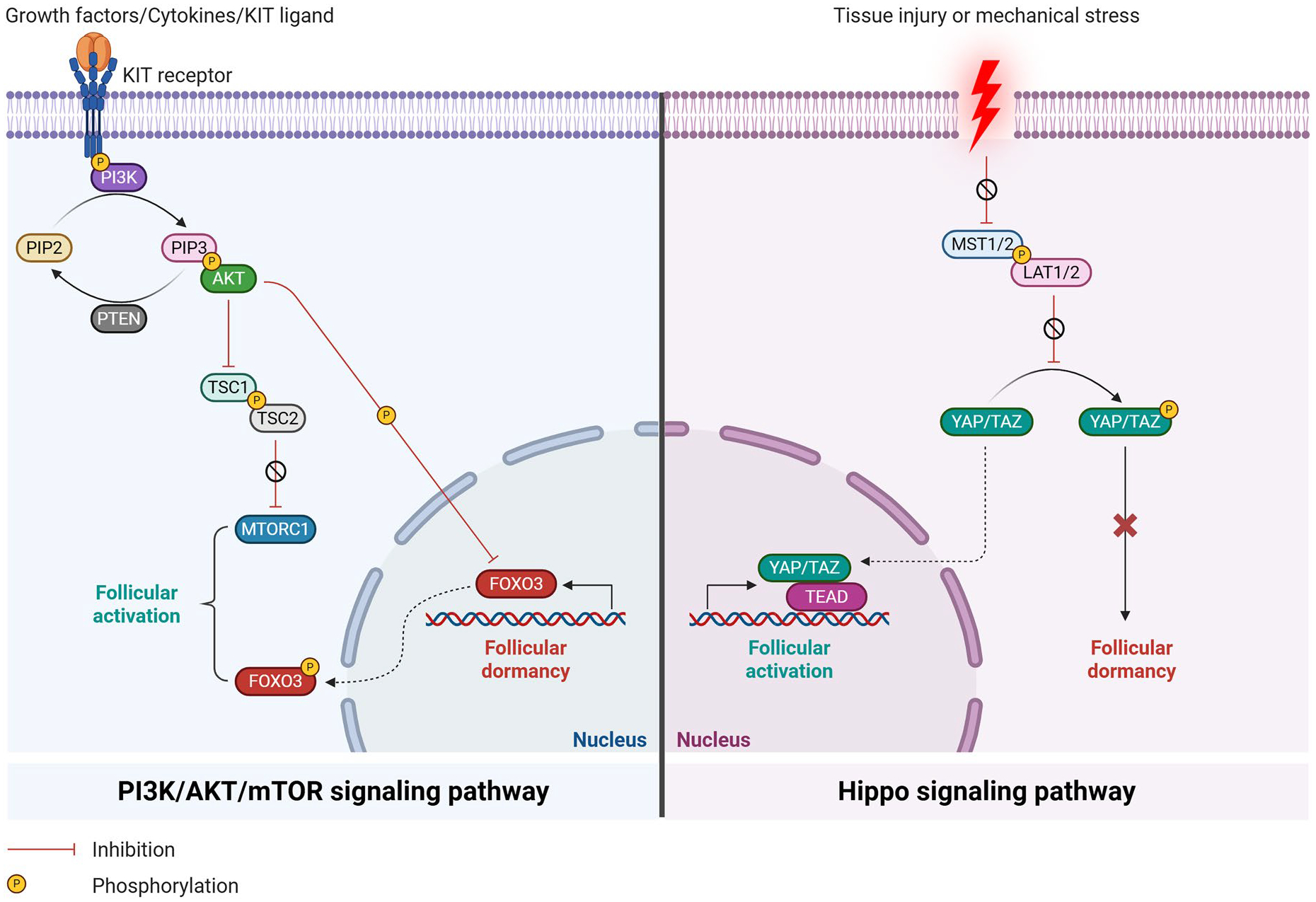
Molecular regulation of oocyte meiotic arrest and resumption. Before the LH surge (left panel): Mural granulosa cells express natriuretic peptide precursor C (NPPC) (encoding C-type natriuretic peptide, CNP) under the influence of FSH, estradiol, and oocyte-secreted factors. Secreted CNP binds natriuretic peptide receptor 2 (NPR2) on cumulus granulosa cells, activating guanylyl cyclase (GC) to convert guanosine triphosphate (GTP) into cyclic guanosine monophosphate (cGMP). cGMP diffuses into oocytes through open gap junctions (GJ), where it inhibits phosphodiesterase 3 A (PDE3A), maintaining elevated cyclic adenosine monophosphate (cAMP) levels. cAMP is also produced endogenously by oocyte adenylyl cyclase (AC) *via* G-protein–coupled receptor (GPCR) signaling. High cAMP levels sustain meiotic arrest at prophase I. After the LH surge (right panel): Luteinizing hormone (LH) binds its receptor (LHR) on theca cells, suppressing NPPC expression and thereby reducing CNP and cGMP levels. LH signaling also triggers GJ closure and activates PDE3A, leading to cAMP degradation. The resulting drop in cAMP relieves meiotic arrest, allowing oocytes to resume meiosis.

In addition to its direct role in folliculogenesis, KIT/KITL signaling is linked to key intracellular pathways such as PI3K and mTORC1 (Figure 2). Upon KITL binding, PI3K is recruited to the oocyte membrane, triggering PIP3-mediated AKT phosphorylation. Activated AKT not only suppresses FOXO3, a known inhibitor of follicle activation, but also stimulates the mTORC1 pathway through phosphorylation of TSC1/2, promoting oocyte growth and follicular progression (Reddy et al. 2005; Sánchez and Smitz 2012; Hannon and Curry 2018).

Along with KIT, germ cells express receptors for other growth factors such as fibroblast growth factors (FGFs), platelet-derived growth factor (PDGF) and cytokines like leukemia inhibitory factor (LIF). Activation of these receptors by their respective ligands (KITL, FGF2, PDGF, and LIF) has been shown to enhance PGC survival, migration, and proliferation *in vitro* (Dolci et al. 1991; Farini et al. 2005; Takeuchi et al. 2005; Yabuta et al. 2006; Gu et al. 2009; Todaro et al. 2019).

#### Hippo signaling

The Hippo pathway regulates follicular growth and maturation by controlling cell proliferation, migration, and differentiation, which are critical for follicular development from primordial to preovulatory stages (Ye et al. 2017; Hu et al. 2019). In the mammalian ovary, Hippo pathway functions through a set of core components involving mammalian STe20-like protein kinase 1/2 (MST1/2) and large tumor suppressor homolog 1/2 (LATS1/2), yes-associated protein (YAP), transcriptional co-activator with PDZ-binding motif (TAZ) and transcriptional enhanced associate domain (TEAD) (Figure 2). When the Hippo pathway is active, MST1/2 triggers LATS1/2 which then phosphorylates YAP and TAZ. This phosphorylation sequesters YAP and TAZ in the cytoplasm and prevents their binding to TEAD, thereby inhibiting follicular development (Pennarossa et al. 2021; Clark et al. 2022). On the contrary, disrupting the Hippo pathway decreases the expression levels of MST1/2 and LATS1/2, allowing YAP and TAZ to enter the nucleus, bind to the TEAD, ending by follicular activation (Varelas 2014; Lv et al. 2019; Tsoi et al. 2019).

### Hormonal regulation

Although granulosa cells of early follicular stages (i.e., primary and secondary follicles) express FSH receptors (O’Shaughnessy et al. 1996; Oktay et al. 1997), primordial to preantral follicle progression is mostly independent of gonadotropins (Oktay et al. 1998; Fortune et al. 2000). Instead, such gonadotropin-independent phase is primarily driven by intraovarian factors secreted from either the oocyte, granulosa cells or both (Table 1). Once follicles progress beyond the preantral stage, FSH dominates. FSH binds to its receptor (FSHR) in granulosa cell, triggering the cyclic adenosine monophosphate (cAMP)-dependent protein kinase A (PKA) signaling pathway. This signaling pathway induces *CYP19A1*, the gene encoding aromatase enzyme, which is necessary for estrogen biosynthesis from theca-derived androgens (the two-cell, two-gonadotropin hypothesis) (Hillier et al. 1994). Estrogen, in turn, upregulates FSHR mRNA expression, supporting FSH-mediated follicular growth and maturation (McGee and Hsueh 2000; Hannon and Curry 2018). In contrast, anti-Müllerian hormone (AMH), secreted by granulosa cells of preantral and early antral follicles, reduces FSH sensitivity in the growing follicles, suppressing excessive follicular recruitment (Fortune 2003). *AMH* Knockdown/knockout causes premature follicle activation and ovarian reserve depletion in mice (Durlinger et al. 2002a; Durlinger et al. 2002b). On the other hand, FSH upregulates BCL-2 mRNA expression in granulosa cells through the PI3K-Akt pathway (Wang et al. 2012). BCL-2, an anti-apoptotic gene, enhances granulosa cell survival and proliferation, thus preventing follicular atresia (Ratts et al. 1995; Rucker et al. 2000). FSH also induces LH/choriogonadotropin receptor (LHCGR) mRNA expression, priming granulosa cells for LH responsiveness (Dierich et al. 1998; Burns et al. 2001).

The processes of follicle selection and dominance are governed by differences in follicular sensitivity to FSH and intra-follicular signaling mechanisms (Son et al. 2011). During the early follicular phase, regression of the corpus luteum from the previous cycle leads to a decline in progesterone levels, thereby permitting a transient rise in circulating FSH. This FSH increase recruits a cohort of antral follicles that grow synchronously in a distinct ‘wave’ (Schipper et al. 1998). Follicle selection occurs when one follicle, typically the largest and possessing the greatest granulosa cell mass, exhibits relatively higher FSH receptor expression and aromatase activity, enabling it to outcompete subordinate follicles for limiting FSH and establish dominance (Irving-Rodgers et al. 2009).

Once selected, the dominant follicle consolidates its position through coordinated intraovarian and systemic regulatory mechanisms (Son et al. 2011). Locally, paracrine factors such as IGF-1 and activin promote granulosa cell proliferation and survival within the selected follicle, thereby enhancing its competitive advantage over subordinate follicles (Fortune et al. 2004; Knight and Glister 2006). Systemically, sustained FSH-stimulated estrogen production, together with inhibin B secretion from granulosa cells, establishes negative feedback at the hypothalamic-pituitary axis. Estradiol, the most active form of follicular estrogens, suppresses GnRH secretion *via* hypothalamic kisspeptin neurons and reduces pituitary responsiveness to GnRH, while inhibin B selectively inhibits FSH release, resulting in the characteristic mid-follicular FSH drop (Findlay and Drummond 1999; Findlay et al. 2000; Dungan et al. 2006; Hannon and Curry 2018). This decline in circulating FSH imposes a survival threshold that reinforces follicle dominance, as only the follicle with the highest gonadotropin sensitivity persists, whereas subordinate follicles undergo atresia (Findlay and Drummond 1999; Baerwald et al. 2012).

As the dominant follicle continues to mature, granulosa cells upregulate LH receptors, enabling a functional shift from FSH to LH dependence that sustains follicular growth despite declining FSH levels. Persistently elevated estradiol concentrations in the preovulatory follicle subsequently reverse hypothalamic-pituitary feedback, triggering the preovulatory LH surge (Bomsel-Helmreich et al. 1979; Moenter et al. 1990; 1992; Herbison 2008). The LH surge initiates a cascade of events, including cumulus expansion, resumption of oocyte meiosis, follicular wall remodeling, and coordinated follicular contractions, culminating in ovulation (Robker et al. 2018; Duffy et al. 2019). Following ovulation, tonic LH stimulation drives luteinization of granulosa and theca cells within the ruptured follicle, leading to corpus luteum formation and progesterone secretion essential for pregnancy establishment and maintenance (Kwintkiewicz and Giudice 2009).

## Oocyte maturation

Oocyte developmental competence refers to the oocyte ability to be fertilized and develop into a healthy embryo that can grow and give live birth at term. To achieve this competence, the oocytes must undergo a gradual process of both nuclear and cytoplasmic maturation (Zuccotti et al. 1998; Sánchez and Smitz 2012; Conti and Franciosi 2018).

### Nuclear maturation

Around the time of birth, oocytes enters a prolonged arrest at the first meiotic prophase stage, also known as germinal vesicle (GV)-stage oocytes (Pei et al. 2023). Non-grown and growing oocytes are characterized by non-surrounded nucleolus (NSN; chromatin is scattered throughout the nucleolar area), whereas fully grown competent oocytes are characterized by surrounded nucleolus (SN; chromatin is highly condensed, forming a ring around the nucleolus). SN oocytes exhibit greater developmental competence, the capacity of oocytes to complete meiosis and produce blastocysts following fertilization, compared to NSN oocytes (Bouniol-Baly et al. 1999; Christians et al. 1999; Yao et al. 2021).

#### Molecular regulation of meiotic arrest

Presence of cAMP, synthesized by adenylyl cyclase, at high levels within the mammalian oocytes is indispensable for maintaining oocyte arrest at prophase-I stage, thereby preventing meiotic resumption (Downs et al. 1989). cAMP primarily comes from the cumulus cells to oocytes though gap junctions. However, oocytes can also endogenously produce low levels of cAMP *via* the activation of G-protein coupled receptors 3 and 12 (GPCR3, GPCR12) (Mehlmann 2005; Vaccari et al. 2008).

Additionally, the influx of cyclic guanosine monophosphate (cGMP) from the cumulus cells to oocytes inhibits cAMP-phosphodiesterase 3 A (PDE3A), the enzyme responsible for cAMP degradation, maintaining high cAMP levels, thereby preventing meiotic resumption (Norris et al. 2009; Vaccari et al. 2009). In such manner, it has been reported that the oocytes themselves significantly contribute to their own meiotic arrest. The oocytes stimulate cumulus cells to express natriuretic peptide receptor 2 (NPR2) which is activated by its ligand C-type natriuretic peptide (CNP); NPR2-CNP interaction is essential for cGMP synthesis (Zhang et al. 2010; 2011). Further studies suggest that endocrine hormones, including FSH, estradiol, and oocyte-secreted factors (OSFs), may enhance this regulatory loop by upregulating natriuretic peptide precursor C (NPPC), CNP encoding gene, and/or NPR2 in cumulus cells, thereby reinforcing the mechanisms that maintain meiotic arrest (Zhang et al. 2010).

#### Molecular regulation of meiotic resumption

The oocytes remain meiotically arrested until LH hits its preovulatory surge. Preovulatory LH surge awakens the oocyte from its dormant state and drives meiosis to progress beyond prophase I. The key regulator of meiotic resumption is the sharp decline in oocyte cAMP levels triggered by preovulatory LH surge. LH promotes PDE3A activation. In addition to its direct activation of PDE3A, LH has the capacity to release the cGMP inhibitory effect on PDE3A activity by limiting cGMP diffusion from the cumulus cells to oocytes in several ways. First, LH induces a massive cumulus expansion which in turn decreases the physical communication between the oocytes and cumulus cells, cGMP precursor. LH also downregulates mRNA expression of NPPC, thus reducing cGMP production in the cumulus cells. As well, LH stimulates the closure of jap junctions between the oocytes and cumulus cells. Altogether, low levels of oocyte cGMP/cAMP, along with high PDE3A activity leads to cAMP degradation and oocyte meiosis progression (Richard et al. 2001; Sela-Abramovich et al. 2005; Norris et al. 2008; Kawamura et al. 2011).

**Figure 3.**
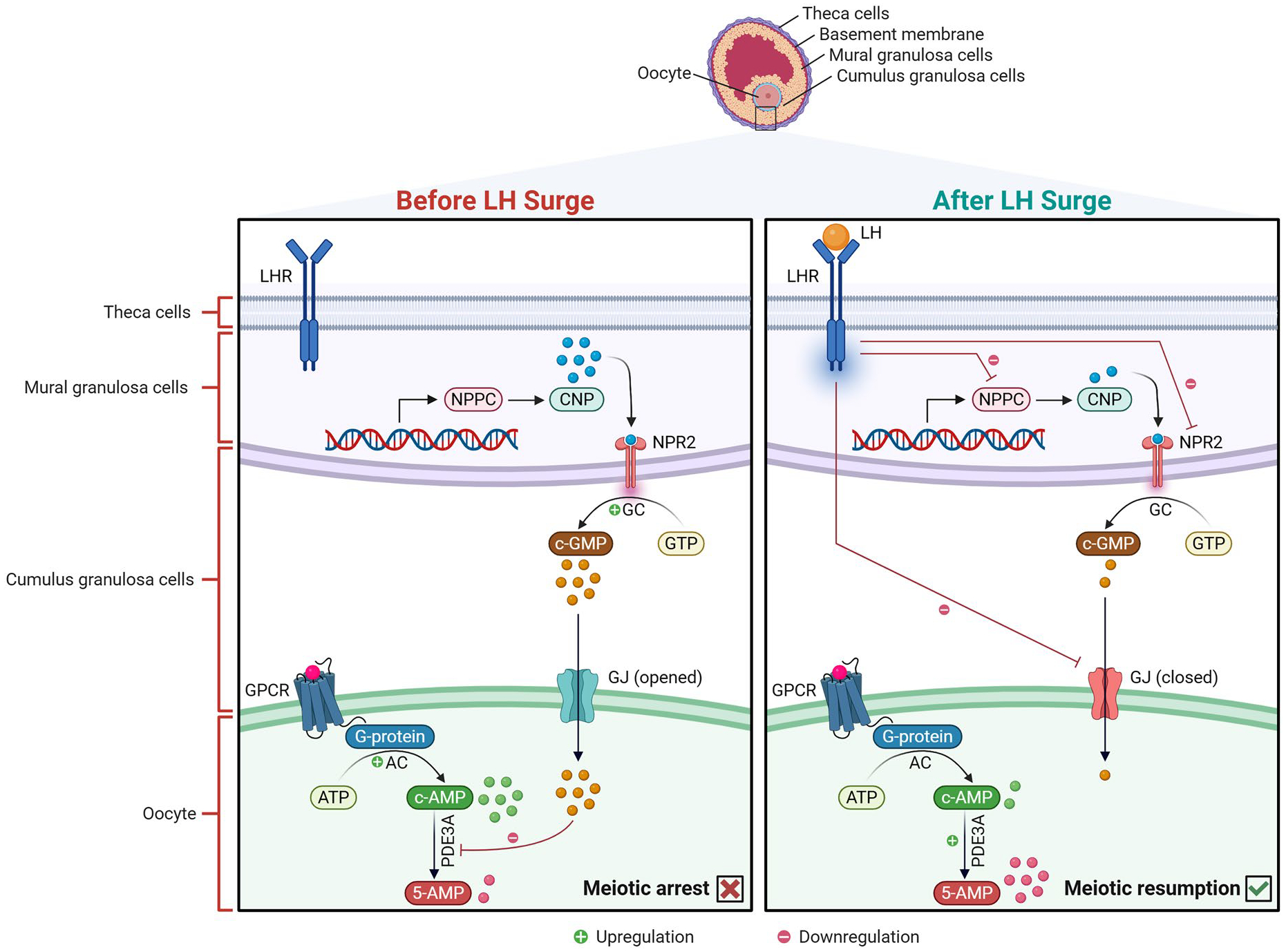
Signaling pathways regulating the balance between follicular dormancy and activation in mammalian ovaries. Left panel: The PI3K/AKT/mTOR pathway is activated by growth factors, cytokines, and KIT ligand (KITL) binding to KIT receptors on the oocyte membrane. KIT/KITL signaling recruits phosphoinositide 3-kinase (PI3K), which converts phosphatidylinositol 4,5-bisphosphate (PIP2) to phosphatidylinositol 3,4,5-trisphosphate (PIP3) and activates protein kinase B (AKT). Activated AKT phosphorylates forkhead box O3 (FOXO3), promoting its cytoplasmic translocation and inactivation, and also phosphorylates tuberous sclerosis complex 1/2 (TSC1/TSC2), which inhibits the mammalian target of rapamycin complex 1 (mTORC1). These events collectively relieve follicular dormancy, enabling oocyte growth and follicular progression. Phosphatase and tensin homolog deleted on chromosome 10 (PTEN) opposes PI3K activity, preventing premature follicle activation. Right panel: In the active Hippo pathway, mammalian STE20-like protein kinases 1/2 (MST1/2) phosphorylate large tumor suppressor homologs 1/2 (LATS1/2), which in turn phosphorylate yes-associated protein (YAP) and transcriptional co-activator with PDZ-binding motif (TAZ). Phosphorylated YAP and TAZ are retained in the cytoplasm, preventing their interaction with transcriptional enhanced associate domain (TEAD) and maintaining follicular dormancy. When the ovary experiences tissue injury or mechanical stress, the Hippo pathway is disrupted, MST1/2 and LATS1/2 activity decreases, allowing YAP and TAZ to translocate into the nucleus, bind TEAD, and promote follicular activation.

The molecular mechanisms regulating meiotic arrest and resumption, including the roles of cGMP, cAMP, and PDE3A, are summarized in Figure 3.

#### Nuclear envelope breakdown and spindle assembly

Upon meiotic resumption, the oocyte undergoes nuclear envelope breakdown (NEBD, also known as GVBD) followed by spindle formation. Spindle assembly is crucial for proper chromosome segregation in both meiotic and mitotic cells (Prosser and Pelletier 2017). In somatic cells, spindle assembly is primarily driven by the major microtubule organizing centers (MTOCs), centrosomes (Doxsey 2001; Petry 2016). Each centrosome, consists of two centrioles surrounded by pericentriolar material, serves for microtubule nucleation and plays a critical step in bipolar spindle formation (Badano et al. 2005; Vertii et al. 2016).

Although centrosomes containing centrioles are present in spermatozoa, they are eliminated from mammalian oocytes during early oogenesis (Manandhar et al. 2005; Mikeladze-Dvali et al. 2012; Pimenta-Marques et al. 2016). Instead, oocytes develop acentriolar MTOCs, which act as hubs for microtubule nucleation and spindle formation spindle (Schuh and Ellenberg 2007; Breuer et al. 2010; Clift and Schuh 2015; Balboula et al. 2016; Wu et al. 2018). According to MTOC dynamics, three distinct classes of MTOCs have been identified in mouse oocytes. In GV-arrested oocytes, MTOCs (class I) are initially localized to the perinuclear area (Szollosi et al. 1972). Just before NBED, these perinuclear MTOCs fragment into smaller MTOCs (Luksza et al. 2013; Clift and Schuh 2015), which then cluster and sort into two groups, ultimately forming a bipolar spindle (Breuer et al. 2010; Balboula et al. 2016). In addition to perinuclear MTOCs, another group of MTOCs has been shown in the oocyte cytoplasm. Some of these cytoplasmic MTOCs (class II) migrate toward the oocyte center, participating in spindle formation (Schuh and Ellenberg 2007; Clift and Schuh 2015). The prevailing view is that microtubules and their associated factors are the only cytoskeletal components driving spindle pole focusing in oocytes. However, recent work demonstrated that spindle-localized F-actin, and their enrichment at MTOCs, promotes MTOC clustering and sorting at the spindle poles, two key steps required for spindle pole focusing (Soto-Moreno et al. 2025). At NEBD, a subgroup of cytoplasmic MTOCs [class III, termed metaphase cytoplasmic MTOCs (mcMTOCs)] appears in the cytoplasm and does not contribute to spindle formation, but plays a critical role in regulating spindle positioning (Londoño-Vásquez et al. 2022). Unlike mouse oocytes, the presence of MTOCs in human oocytes remains controversial. It has long been argued that human oocytes lack MTOCs at the spindle poles (Holubcová et al. 2015; So et al. 2022). However, a recent study identified a new population of microtubule organizing centers (termed huoMTOC), which fragments and subsequently localizes to kinetochores, promoting microtubule nucleation and spindle assembly (Wu et al. 2022). On the other hand, both mitotic and meiotic cells can utilize additional pathways for spindle assembly. Among them, RanGTP (Ras-like nuclear promoted by guanosine triphosphate) pathway is well characterized (Khodjakov et al. 2000; Basto et al. 2006; Cesario and McKim 2011; Holubcová et al. 2015). RanGTP forms a gradient around chromosomes. Chromosomes-mediated RanGTP gradient triggers the dissociation of spindle assembly factors (SAFs) from their inhibitory binding to importins. Once activated, SAFs initiate microtubule nucleation and spindle formation (Gruss et al. 2001; Nachury et al. 2001; Dumont et al. 2007; Meunier and Vernos 2016). Augmin complex and chromosomal passenger complex (CPC) pathways have been also involved in spindly assembly in Xenopus (Colombié et al. 2013) and Drosophila oocytes (Radford et al. 2012; Das et al. 2016), whereas Polo like kinase 4 (Plk4) and Aurora Kinase A cooperate in bipolar spindle assembly in mouse oocytes (Bury et al. 2017).

#### Spindle positioning and migration

Since the spindle forms at the site of NEBD, the position of oocyte nucleus (GV) is a major determinant of the initial spindle position (Kincade et al. 2023). Unlike human (Levi et al. 2013), bovine (Fair et al. 1997), and porcine (Lee et al. 2000) oocytes, which typically exhibit a peripherally located nucleus, the nucleus is centrally located in the majority of mouse oocytes (Brunet and Maro 2007), and therefore, the spindle is initially formed at the oocyte center in mice (Kincade et al. 2023). Interestingly, in both human and mice, central nucleus positioning correlates with greater maturation rates when compared to oocytes with a peripheral nucleus (Levi et al. 2013). Given that the spindle position defines the cell division plane (McCarthy and Goldstein 2006), the central spindle must migrate to the oocyte periphery (cortex) to allow asymmetric division and small polar body extrusion (Longo and Chen 1985). This asymmetric division is very critical to ensure that the oocyte retains most of the cytoplasmic reserves of maternal RNAs and proteins required for early embryonic development (Verlhac et al. 2000; Schuh and Ellenberg 2008). F-actin, and its regulatory molecules, is the main cytoskeletal component driving spindle positioning and migration. Disrupting F-actin impairs spindle migration leading to a more symmetrical division with emergence of a large polar body (Azoury et al. 2008; Li et al. 2008; Schuh and Ellenberg 2008). A recent report showed that initial positioning of spindle at the oocyte center is crucial to avoid the premature exposure to cortical CDC42 signaling, leading to excessive tubulin tyrosination (Kincade et al. 2023). In contrast to basal tubulin tyrosination, excessive tubulin tyrosination perturbs kinetochore-microtubule (K-MT) attachments, resulting in the development of aneuploid eggs (Kincade et al. 2023). Thus, spindle positioning and migration within the oocyte must be tightly and timely regulated. In such manner, it has been shown that mcMTOCs-nucleated microtubules anchor the spindle to the oocyte cortex, thereby generating an opposing force against F-actin-derived spindle migration (Londoño-Vásquez et al. 2022). The balance between these two forces ensures the initial positioning of the spindle at the oocyte center and regulates the timing of spindle migration (i.e., after establishing correct K-MT attachments) (Londoño-Vásquez et al. 2022).

### Cytoplasmic maturation

Oocyte developmental competence is primarily dependent on the oocyte cytoplasmic maturation (Zuccotti et al. 1998; Sánchez and Smitz 2012; Conti and Franciosi 2018). Cytoplasmic maturation encompasses a series of complex events including reorganization of cytoplasmic organelles, RNA storage and protein synthesis, as well as epigenetic modifications (Mao et al. 2014; Coticchio et al. 2015).

#### Reorganization of cytoplasmic organelles

##### Mitochondria.

Various processes are involved in oocyte cytoplasmic maturation processes such as organellar movement, cytoskeletal dynamics, RNA storage, and protein synthesis require a high demand of adenosine triphosphate (ATP), the usable form of energy within cells. Mitochondria, the energy powerhouses in all body cells including oocytes, play a crucial role in meeting this ATP demand (Stojkovic et al. 2001). For proper oocyte maturation, mitochondria relocate to regions with high energy demand. In the immature oocytes, mitochondria cluster around the perinuclear area (GV), while the subcortical region is devoid from mitochondria. As the oocyte undergoes GVBD and upon spindle formation, mitochondria begin to enrich around the spindle and spread throughout the ooplasm, achieving their maximal distribution at Metaphase II (MII) stage (Dumollard et al. 2006; Yu et al. 2010).

Alongside mitochondrial redistribution during oocyte maturation, the number of mitochondria in mammalian oocytes significantly increases throughout development. Early germ cells contain approximately 10 mitochondria, which rise to 200 at the oogonium stage. Primary oocytes house around 6,000 mitochondria, and by the completion of oocyte maturation, mitochondrial DNA (mtDNA) copy numbers exceed 100,000, with each mitochondrion carrying one or two copies (Ferreira et al. 2009). Since mtDNA does not replicate during the early embryonic stages, the high mtDNA copy number in mature oocytes ensures a sufficient supply of mitochondria until implantation (Shoubridge and Wai 2007). Consequently, inhibiting mtDNA replication *via* 2’,3′-dideoxycytidine, a nucleoside analog that blocks mtDNA replication, does not affect oocyte maturation or mitochondrial redistribution, but significantly reduces blastocyst formation in mice (Ge et al. 2012; Mao et al. 2012).

##### Golgi apparatus.

In mammalian oocytes, Golgi apparatus is indispensable for a variety of intracellular trafficking processes involved in protein transport, storage, and metabolism (Mao et al. 2014). In GV oocytes, Golgi apparatus appears as a membranous organelle dispersed throughout the ooplasm with a mild preference at the oocyte center. Upon NEBD, Golgi apparatus fragments into small structures, which accumulate at the oocyte center (Moreno et al. 2002).

Using a mouse oocyte model, it has been shown that treatment of GV oocytes with brefeldin A, a membrane-trafficking inhibitor that disturbs Golgi apparatus function, prevents NEBD and disrupts spindle assembly with a subsequent defective oocyte maturation (Moreno et al. 2002). However, brefeldin A treatment post-NEBD does not affect embryo cleavage (Payne and Schatten 2003), suggesting that Golgi apparatus is only required during NEBD, but once NEBD is accomplished, Golgi apparatus is no longer needed for further embryonic development.

##### Endoplasmic reticulum.

The endoplasmic reticulum (ER), the main internal reservoir of calcium ions, plays an important role in lipid and protein metabolism within the cells (Bravo et al. 2013). During oocyte maturation, ER undergoes significant biochemical and ultrastructural changes. While ER is uniformly distributed throughout the cytoplasm in GV oocytes, it forms tiny clusters of 1–2 μm that migrates and localizes to the cortical regions by MII. After fertilization, ER clusters disappear and do not reappear in early cleavage-stage embryos (Mehlmann et al. 1995; Kline 2000; FitzHarris et al. 2007).

Such biochemical and ultrastructural changes ensure proper regulation of the intracellular calcium ions (Ca^+2^) (Gomez-Fernandez et al. 2012). One of the main roles played by Ca^+2^ in oocyte biology is to prevent polyspermy incidence either through fast or slow block (Machaca 2007; Ullah et al. 2007). The fast block occurs when a rise in Ca^+2^ at fertilization gates activates chloride (Cl^−^) channels, which in turn depolarize oocyte membrane preventing further sperm entry. This model is clearly demonstrated in *Xenopus*, in which oocyte-sperm fusion is voltage-dependent (Jaffe et al. 1983).

The slow block to polyspermy is induced by Ca^+2^ -mediated cortical reaction (Wolf 1974; Schuel 1978). In GV oocytes, the cortical granules, minute aggregates measuring 0.2 to 0.6 μm in diameter, are initially distributed in the cytoplasm (Gulyas 1980; Gross et al. 2000). Later, the cortical granules migrate to the sub-cortical regions in MII oocytes. Upon fertilization, cortical granules undergo exocytosis, releasing their enzymatic contents into the perivitelline space to prevent polyspermy by degrading sperm-binding receptors (Yoshida et al. 1993; Hosoe and Shioya 1997).

#### RNA storage and protein synthesis

##### Transcriptional silencing.

Oocyte growth, approximately from 20 to 80 μm in mice and from 35 to120 μm in human (Eppig and O’Brien 1996; Picton et al. 1998), is accompanied by a high rate of synthesis and accumulation of RNA and protein which are crucial for successful oocyte maturation and the subsequent embryo development (Moore and Lintern-Moore 1978; Bachvarova 1985; Eichenlaub-Ritter and Peschke 2002). RNA transcription hits the peak during the early stages of development, coinciding with follicular activation and cell proliferation. However, by the end of growth phase (i.e., fully grown oocytes), transcription is silenced, and RNA storage and degradation become the prevalent processes (Bachvarova et al. 1985; De La Fuente et al. 2004; Su et al. 2007).

##### Storage of maternal mRNA.

The total RNA in fully-grown meiotically competent mouse oocytes has been estimated to be around 6 ng, nearly 200 folds the amount present in a typical somatic cell (Sternlicht and Schultz 1981). The majority (~ 65%) of oocyte RNA consists of ribosomal RNA, while only about 10–15% is heterogenous RNAs (Wassarman and Kinloch 1992). The fate of oocyte mRNA is mainly determined by its binding with various proteins; these proteins control the mRNA’s accessibility to translation initiation factors and ribosomes, thereby detecting if it will be immediately translated into protein or stored for later use (Eichenlaub-Ritter and Peschke 2002).

Within the oocyte, maternal mRNAs are stored in ribonucleoprotein (RNP) complexes to protect them from degradation. In addition to mRNA role in translation, RNA plays important roles in regulating CPC function during oocyte meiosis in translation independent manner (Balboula et al. 2017). Recently, it has been shown that mammalian oocytes develop a membraneless structure containing RNPs and mitochondria, as a storage site for maternal mRNAs (Cheng et al. 2022). These stored mRNAs undergo a polyadenylation process, in which a short poly(A) tail is added to the 3′ end of mRNA. This process stabilizes the mRNA and promotes their translation, ensuring the mRNA availability for further steps of oocyte and embryo development (de Moor and Richter 2001).

##### Degradation of maternal mRNAs.

Maternal mRNAs are subjected to degradation process during oocyte maturation and early embryo development (Oh et al. 2000). Poly(A) tail shortening, known as de-adenylation, is a key regulator of oocyte mRNA degradation. The poly(A) tails of certain mRNAs are selectively shortened, marking them for degradation (Ozturk and Uysal 2017). This process helps in the removal of unnecessary mRNAs, thereby improving the oocyte microenvironment for activation of stored or newly transcribed mRNAs (Su et al. 2007).

Also, de-adenylation process plays a significant role in activating maternal-to-zygotic transition (MZT), in which regulation of gene expression is taken over by zygotic genome instead of maternal mRNAs (Sha et al. 2019). In mice, MZT occurs at the two-cell stage embryos. By this stage, about 90% of stored maternal mRNAs should be degraded, allowing early embryo to begin its own transcriptional activity (Schellander et al. 2007). De-adenylation is the first and rate-limiting step for the efficient removal of maternal mRNAs, ensuring a timely MZT activation (Walser and Lipshitz 2011).

#### Epigenetic modifications

The growing oocytes undergo a variety of posttranslational modifications such as acetylation, methylation, phosphorylation, glycosylation, ubiquitination, and SUMOylation of different proteins (He et al. 2021; Bi et al. 2023). These modifications suggest that epigenetic regulation plays a significant role during the oocyte maturation process (Li 2002; Wu et al. 2021; Wasserzug Pash et al. 2022; Bozdemir et al. 2025).

##### Histone acetylation.

Histone acetyl transferases (HATs) and histone deacetylases (HDACs) regulate lysine acetylation of histones (Gallinari et al. 2007). Acetylation of H3/4 stimulates open chromatin configuration, enhancing transcriptional activity, while histone deacetylation compacts chromatin and inhibits mRNA transcription (Liu et al. 2011). During oocyte maturation, histones are deacetylated, and perturbing this deacetylation leads to meiotic defects and aneuploidy (Kim et al. 2003; Akiyama et al. 2004; Ma et al. 2012; Ma and Schultz 2013; Balboula et al. 2014, 2015; Ma and Schultz 2016).

##### Histone methylation.

One of the main factors influencing chromatin activity is histone methylation, which is controlled by histone lysine methyl transferases (KMTs) and histone lysine demethylases (KDMs) (Sha et al. 2020). While H3K4 methylation activates chromatin, H3K9 or H3K27 methylation results in chromatin inactivation (Werner and Ruthenburg 2011). In developing oocytes, chromatin transition from NSN to SN type is associated with high levels of H3K4 methylation type (Yu et al. 2017). Of known that, the SN-oocytes have higher developmental competence compared to the NSN ones (Ma et al. 2013; Zhang et al. 2019).

##### Histone phosphorylation.

Serine, threonine, or tyrosine residues are the most common sites for protein phosphorylation, which controls cell cycle related activities in a variety of signal transduction pathways (Schatten and Sun 2014). For example, chromatin condensation of either mitotic or meiotic cells can be negatively impacted by histone H3 phosphorylation at Ser10 and Ser28 (Houben et al. 2005; Swain et al. 2007). Histone H3 is phosphorylated at Ser28 in mitotic cells by Aurora kinase B, the catalytic subunit of the CPC. Mammalian oocytes contain Aurora kinase C which shares high sequence homology with Aurora kinase B. Inhibition of Aurora kinase B and/or C activity decreases H3S10 phosphorylation and results in chromosome misalignment, abnormal K-MT attachments, perturbed spindle assembly checkpoint and cytokinesis defects (Schindler et al. 2012; Balboula and Schindler 2014; Quartuccio and Schindler 2015).

##### Histone glycosylation.

A wide range of glycosylated proteins including FSH, LH, GDF9, BMP15, and AMH play essential roles in female reproductive biology. Protein glycosylation, one of the most common post-translational modifications impacting protein metabolism and function, occurs in two forms: N-linked and O-linked glycosylation (Ohtsubo and Marth 2006). N-linked glycosylation is much more important in oocyte growth and maturation than O-linked glycosylation. Dolichyl-phosphate alpha-N-acetyl-glucosaminyl-phosphotransferase (DPAGT1), an enzyme involved in N-linked glycosylation, is crucial for follicular development. Additionally, *DPAGT1* mutation leads to subfertility in mice due to the emergence of a thin and fragile zona pellucida (ZP) which in turn causes poor developmental competence post-fertilization (Li H et al. 2020).

##### Histone ubiquitination.

In addition to its well-known functions in protein metabolism, cell cycle control, and mRNA expressions (Bassermann et al. 2014), the ubiquitin proteosome system (UPS) is also involved in oocyte maturation process (Susor et al. 2010). Anaphase-promoting complex (APC), through its E3 ubiquitin ligase activity, ubiquitinates securin and cyclin B1 targeting them for degradation by the proteasome (Jones 2011). Such degradation triggers the oocyte transition from metaphase I to anaphase stage (Huo et al. 2004). Besides, cullin ring-finger ubiquitin ligase 4 (CRL4), along with its adaptor molecule, DDB1 and CUL4-associated factor 13 (DCAF13), target PTEN for degradation, thereby stimulating oocyte meiosis resumption. *DCAF13* depletion results in oocyte arrest at prometaphase I, emphasizing its vital role in oocyte maturation (Zhang et al. 2020). On the other hand, mammalian oocytes express deubiquinating enzymes such as ubiquitin C-terminal hydrolases1 (UCHL1) and UCHL3. While UCHL1 expression is enriched in the oocyte cortex, UCHL3 localizes to the spindle. Interfering with UCHL activity impairs spindle formation associated with chromosome misalignment (Mtango et al. 2012; 2014). Moreover, it has been reported that UPS regulates extracellular matrix deposition and steroidogenesis during cumulus cell expansion, suggesting its contribution to follicular development (Nagyova et al. 2012).

##### Histone SUMOylation.

One of the most important post-translational modifications required for oocyte maturation is the SUMOylation/de-SUMOylation process (Wang et al. 2010). This process involves the addition or removal of small ubiquitin-related modifier (SUMO1, SUMO2, and SUMO3) or ubiquitin conjugating enzyme E2 I (UBE2I) at lysine residues (Schatten and Sun 2014). The localization of SUMO proteins in mammalian oocytes differs according to meiotic stage. In GV-arrested oocytes, SUMO1 is expressed in the nuclear membrane while SUMO2/3 is detected in the nucleoplasm. With meiotic progression, SUMO1 migrates to the spindle poles whereas SUMO2/3 localize to the centromeres (Yuan et al. 2014; Feitosa et al. 2018). On the other hand, UBE2I is predominantly expressed in the nucleoplasm of GV-arrested oocytes, then gradually declines post-GVBD (Ihara et al. 2008). It has been reported that inhibiting SUMOylation process in GV-arrested oocytes significantly impairs NEBD and meiotic progression. Moreover, the oocytes that progressed beyond the GV stage showed disrupted K-MT attachments, abnormal spindle formation, chromosome misalignment, and higher aneuploidy rates (Yuan et al. 2014; Rodriguez et al. 2019). Overexpression of de-SUMOylation proteins, SUMO-specific proteases (SENP), in oocytes exhibits similar findings, supporting the role of SUMOylation in oocyte maturation (Wang et al. 2010).

## Oocyte-cumulus cells interaction

Throughout folliculogenesis and oogenesis, oocytes engage in bidirectional communication with their surrounding granulosa and cumulus cells. This communication occurs through either paracrine signaling or gap junctions between oocytes and cumulus cells (Russell et al. 2016). Regulation of oocyte-cumulus cells interaction is crucial for follicular growth, oocyte maturation, and early embryonic development (Wigglesworth et al. 2013). Within the antral follicle, the cumulus cells closely surround and connect to oocytes *via* gap junctions of cytoplasmic projections penetrating the oocyte zona pellucida (i.e., transzonal projections; TZPs), developing a structure called cumulus-oocyte complexes (COCs) (Kidder and Mhawi 2002). These gap junctions, mediated by connexin 37 and 43, allow the transfer of small molecules between the cumulus cells and oocytes, controlling oocyte growth, maturation and developmental competence (Gittens and Kidder 2005; Winterhager and Kidder 2015).

### Contribution of cumulus cells to oocyte functions

In addition to their well-established role in maintaining meiotic arrest at the prophase I stage, as previously discussed, cumulus cells provide essential metabolic support for oocyte growth and maturation and offer critical protection against oxidative stress (Martinez et al. 2023).

#### Oocyte growth and maturation

Oocytes lack the capacity to efficiently metabolize glucose. Glucose is a key metabolite in energy production required for the whole folliculogenesis and oogenesis stages (Sutton-McDowall et al. 2010). Therefore, oocytes completely rely on cumulus cells for glycolysis to generate glucose metabolites, pyruvate, and lactate. These metabolized can be subsequently utilized by oocytes for ATP production (Funahashi et al. 2008). Similar to glucose metabolism, oocytes have a limited ability to synthesize cholesterol due to the absence of enzymes involved in cholesterol biosynthesis pathway (e.g., MvK, Pmvk, Fdps, Sqle, Cyp51, Sc4mol, and Ebp). On the contrary, cumulus cells not only express these enzymes, but also supply cholesterol precursors to oocytes, helping in cholesterol production (Pratt 1982; Comiskey and Warner 2007). Beyond its well-known function in energy production during oocyte maturation, cholesterol is also indispensable for preimplantation early embryo development (Su et al. 2008). A third example of the metabolic cooperation between oocytes and cumulus cells is amino acid uptake. In comparison to cumulus cells, oocytes exhibit poor amino acid absorbing ability. For instance, *in vitro* matured denuded oocytes (i.e., without cumulus cells) exhibit significantly lower L-alanine levels when compared to COCs (i.e., with cumulus cells) (Eppig et al. 2005). Such finding is referred to the fact that L-alanine is primarily transported by solute carrier family 38, member 1 (SLC38A1). SLC38A1 is absent in oocytes, but it is highly expressed in cumulus cells. Accordingly, cumulus cells play a crucial role in amino acid uptake and protein synthesis, needed for oocyte cytoplasmic maturation events (Colonna and Mangia 1983).

#### Oocyte resilience to oxidative stress

Reactive oxygen species (ROS) are naturally occurring in healthy follicles during physiological processes of folliculogenesis and oogenesis (Goud et al. 2008). Oxidative phosphorylation, the main pathway for energy production within oocytes, may generate ROS as a by-product. However, excessive accumulation of ROS causes a damage to the cellular components including DNA, thereby perturbing oocyte quality and developmental competence (Almansa-Ordonez et al. 2020).

Oxidative stress occurs when ROS levels overwhelm the intracellular antioxidant capacity (Almansa-Ordonez et al. 2020). Since oocytes have a limited antioxidant capacity, cumulus cells play a significant role in oocyte protection from oxidative stress hazards (Tatemoto et al. 2000; von Mengden et al. 2020). One of the most important antioxidants protecting against peroxides is reduced glutathione (GSH). Nicotinamide adenine dinucleotide phosphate (NADPH) helps in recycling oxidized glutathione (GSSG) back to its reduced form (GSH), maintaining its antioxidant activity (Meister 1983). Although oocytes can produce some GSH, their antioxidant capacity mainly depends on GSH and NADPH provided by cumulus cells (Geshi et al. 2000; Luberda 2005). Notably, GSH supplementation enhance cryotolerance of denuded oocytes, highlighting its protective role in the absence of cumulus cells (Trapphoff et al. 2016). Alongside, cumulus cells are capable of production of antioxidant enzymes including catalase (CAT) (El Mouatassim et al. 1999), superoxide dismutase (SOD) (Matos et al. 2009), and glutathione-S-transferases (GSTs) (Hayes and Pulford 1995) at significantly higher levels than oocytes. Altogether, cumulus cells greatly contribute to oocyte antioxidant capacity.

### Contribution of oocytes to cumulus cell function

Currently, there is a consensus on the involvement of OSFs in regulating granulosa/cumulus cell functions. Among these OSFs, GDF9 is essential for granulosa cell proliferation (Elvin and Matzuk 1998; Orisaka et al. 2006). Mice lacking *Gdf9* are infertile due to arrested folliculogenesis at the primary follicle stage (Dong et al. 1996; Carabatsos et al. 1998), while supplementation of *in vitro* maturation (IVM) medium with recombinant GDF9 improves the developmental competence of mouse (Yeo et al. 2008), bovine (Hussein et al. 2006), and porcine (Gomez et al. 2012) oocytes. Importantly, it has been shown that GDF9 synergistically works with BMP15 to promote ovarian functions (Yan et al. 2001). Oocytes-derived BMP15 stimulates granulosa cell differentiation, enhances gonadotropins responsiveness, and mediates steroidogenesis activity (Otsuka et al. 2000). Although *Bmp15* knockout mice exhibit a normal follicular dynamic until antral development, they are sub-fertile due to reduced ovulation and fertilization rates (Yan et al. 2001). In addition, FGFs are another growth factor family produced by mammalian oocytes (Valve et al. 1997; Buratini et al. 2005). Among them, FGF8 works with BMP15 to induce mRNA expression of glycolytic enzymes in cumulus cells, thereby enhancing their glycolytic activity (Sugiura et al. 2007).

### Clinical significance

Recent advances in understanding the molecular mechanisms underpinning oocyte-cumulus cell interaction have extensively improved assisted reproductive technologies (ARTs) (Fair and Lonergan 2023). One significant application is the optimization of IVM culture system through OSFs supplementation, which enhances oocyte quality and supports early embryo development (Martinez et al. 2023). Furthermore, identifying novel molecular biomarkers in the cumulus cells offers a noninvasive promising approach for evaluating oocyte quality and predicting embryo developmental competence (Balboula et al. 2010b, 2010a, 2013, 2020; Li J et al. 2020; Ezz et al. 2024). Besides, a better understanding of OSF-regulated signaling pathways opens new insights into upgrading ovarian stimulation protocols, decreasing the risks of ovarian hyperstimulation syndrome, and enhancing fertility outcomes (Aboulghar et al. 1997; Fábregues et al. 2004; Ishikawa-Yamauchi et al. 2024).

## Conclusion

In conclusion, this chapter outlines the complex cellular and molecular mechanisms underlying folliculogenesis and oogenesis, indicating that female fertility critically depends on the sophisticated molecular dialogue between the oocyte and its surrounding somatic cells, as well as hormonal regulation by the hypothalamic-pituitary-ovarian axis. The key findings emphasize the bidirectional communication between the oocyte and cumulus cells. Cumulus cells nurture the oocyte by providing essential nutrients, RNAs and energy substrates necessary for oocyte growth, developmental competence and protecting it from oxidative stress. In return, the oocyte regulates cumulus cells through the secretion of signaling molecules such as GDF9, and FGFs. This dynamic interplay creates a tightly coordinated microenvironment that is indispensable for both nuclear and cytoplasmic maturation of the oocyte, ultimately ensuring its developmental competence and successful post-fertilization progression.

Oocyte quality and developmental competence are threatened by multiple endogenous and exogenous factors that significantly impact female fertility (Fidler and Bernstein 1999; Sun et al. 2019). Endogenously, genetic mutations affecting key regulators of oocyte growth and development can severely compromise oocyte quality and embryo viability, often resulting in miscarriage and infertility problems (Cai et al. 2013; Loeuillet et al. 2022). Aging further exacerbates these risks by causing mitochondrial dysfunction, epigenetic alterations, and increased aneuploidy, collectively diminishing the pool of developmentally competent oocytes (Balasch 2010; Crawford and Steiner 2015). Exogenously, environmental factors such as heat stress and exposure to a wide array of toxicants, including heavy metals, cigarette smoke, pesticides, plastic compounds, pharmaceuticals, and personal care products, can disrupt oocyte biology in several ways (Hansen 2009; Wang et al. 2016; Mourikes and Flaws 2021; Yao et al. 2023). These involve inducing oxidative stress by increasing reactive oxygen species production, which leads to mitochondrial dysfunction, DNA damage, and apoptosis (Jia et al. 2015; Cheng et al. 2019). They also perturb cytoskeletal organization, resulting in abnormal spindle morphology and chromosome misalignment in developing oocytes (Xu et al. 2020; Wang et al. 2022). Additionally, they interfere with epigenetic regulation, disturbing gene expressions essential for proper oocyte development (Strazzullo and Matarazzo 2017; Menezo et al. 2019). Future research should prioritize deeper investigation into understanding how endogenous and exogenous factors disrupt oogenesis and folliculogenesis, thereby identifying precise therapeutic targets for infertility treatment. Moreover, discovering novel biomarkers capable of specifically detecting damage caused by intrinsic or extrinsic threats would hold significant value for developing potential strategies for improving the quality and developmental competence of female gametes.

## Figures and Tables

**Table 1.** List of transcription factors involved in the regulation of folliculogenesis process.

Gene	Source	Mouse knockout phenotype	Reference
*Phase I: PGCs migration and proliferation*			
*Blimp1 (Prdm1)*	Germ cells	Early PGCs depletion	(Ohinata et al. 2005)
*Prdm14*	Germ cells	Early PGCs depletion	(Yamaji et al. 2008)
*Pou5f1 (Oct4)*	Germ cells	Early PGCs depletion	(Kehler et al. 2004)
*Nanog*	Germ cells	Early PGCs depletion	(Yamaguchi et al. 2009)
*Phase II: Gonadal differentiation & ovarian development*			
*Gata4*	Somatic cells	Gonadal agenesis due to PGCs loss	(Tevosian et al. 2002)
*Sf1 (Nr5a1)*	Somatic cells	Gonadal agenesis due to PGCs loss	(Luo et al. 1994)
*Emx2*	Somatic cells	Gonadal agenesis due to PGCs loss	(Miyamoto et al. 1997)
*Lhx9*	Somatic cells	Gonadal agenesis due to PGCs loss	(Birk et al. 2000)
*Zglp1 (GLP1)*	Somatic cells	Gonadal agenesis due to PGCs loss	(Li et al. 2007; Strauss et al. 2011)
*Wt1*	Somatic cells	Gonadal agenesis due to PGCs loss	(Wilhelm and Englert 2002)
*Wnt4*	Somatic cells	Gonadal agenesis due to PGCs loss	(Manuylov et al. 2008)
*Foxl2*	Somatic cells	Gonadal agenesis due to PGCs loss	(Schmidt et al. 2004; Uda et al. 2004)
*Phase III: Formation and activation of primordial follicles*			
*Figla*	Germ cells	Failure of primordial follicle formation	(Soyal et al. 2000; Joshi et al. 2007)
*Sohlh1*	Oocytes	Impaired primordial to primary follicle transition	(Pangas et al. 2006)
*Sohlh2*	Oocytes	Impaired primordial to primary follicle transition	(Choi et al. 2008; Toyoda et al. 2009)
*Nobox*	Oocytes	Impaired primordial to primary follicle transition	(Rajkovic et al. 2004)
*Lhx8*	Oocytes	Impaired primordial to primary follicle transition	(Choi et al. 2008)
*Phase III: Primary to preantral follicle transition*			
*Taf4b*	Oocytes/Granulosa	Impaired primary to secondary follicle transition	(Falender et al. 2005; Voronina et al. 2007)
*Tbp2 (Trf3)*	Oocytes	Impaired primary to secondary follicle transition	(Gazdag et al. 2007)

## Abbreviations:

AMH: Anti-Müllerian hormone
APC: Anaphase-promoting complex
ARTs: Assisted reproductive technologies
BCL-2: B-cell lymphoma 2
BMP: Bone morphogenetic protein
BLIMP1: B-lymphocyte-induced maturation protein 1
CAT: Catalase
cAMP: Cyclic adenosine monophosphate
CNP: C-type natriuretic peptide
CPC: Chromosomal passenger complex
CRL4: Cullin ring-finger ubiquitin ligase 4
cGMP: Cyclic guanosine monophosphate
DCAF13: DDB1 and CUL4-associated factor 13
DPAGT1: Dolichyl-phosphate alpha-N-acetyl-glucosaminyl-phosphotransferase
EMX2: Empty spiracles homeobox 2
ER: Endoplasmic reticulum
FGFs: Fibroblast growth factors
FIGLA: Factor in the germline alpha
FOXO3: Forkhead box O3
FSH: Follicle stimulating hormone
GATA4: GATA binding protein 4
GDF9: Growth differentiation factor 9
GHSH: Reduced glutathione
GLP1: Germ line transcription factor 1
GPCR3: G-protein coupled receptor 3
GPCR12: G-protein coupled receptor 12
GSTs: Glutathione-S-transferases
GV: Germinal vesicle
GVBD: Germinal vesicle breakdown
HATs: Histone acetyl transferases
HDACs: Histone deacetylases
IGF: Insulin-like growth factor
IVM: In vitro maturation
KDMs: Histone lysine demethylases
KIT: Receptor tyrosine kinase
KITL: Kit ligand
KMTs: Histone lysine methyl transferases
LATS: Large tumor suppressor homolog
LH: Luteinizing hormone
LHCGR: LH/choriogonadotropin receptor
LHX8: LIM homeobox 8
LHX9: LIM homeobox 9
LIF: Leukemia inhibitory factor
MI: Metaphase I
MII: Metaphase II
MST: Mammalian STe20-like protein kinase
mTORC1: Mammalian target of rapamycin complex 1
mtDNA: Mitochondrial DNA
MZT: Maternal-to-zygotic transition
NANOG: Nanog homeobox
NEBD: Nuclear envelope breakdown
NOBOX: NOBOX oogenesis homeobox
Npr2: Natriuretic peptide receptor 2
NR5A1: Nuclear receptor subfamily 5 group A member 1
NSN: Non-surrounded nucleolus
OCT4: Octamer-binding transcription factor 4
OSFs: Oocyte-secreted factors
PDE3A: Phosphodiesterase 3A
PDGF: Platelet-derived drowth factor
PGCs: Primordial germ cells
PI3K: Phosphatidyl-inositol 3-kinase
PKA: Protein kinase A
Plk4: Polo like kinase 4
PRDM1: PR domain zinc finger protein 1
PRDM14: PR domain zinc finger protein 14
POU5F1: POU class 5 homeobox 1
PTEN: Phosphatase and tensin homolog
RNP: Ribonucleoprotein
ROS: Reactive oxygen species
SAFs: Spindle assembly factors
SCF: stem cell factor
SF1: Steroidogenic factor 1
SN: Surrounded nucleolus
SOD: Superoxide dismutase
SLC38A1: Solute carrier family 38, member 1
SOHLH1: Spermatogenesis and oogenesis specific basic helix-loop-helix 1
SUMO: Small ubiquitin-related modifier
TAF4B: TATA-box binding protein associated factor 4B
TAZ: Transcriptional co-activator with PDZ-binding motif
TBP2: TATA-box binding protein 2
TEAD: Transcriptional enhanced associate domain
TGF-β: Transforming growth factor-beta
TRF3: TATA-related factor 3
TZPs: Transzonal projections
UBE2I: Ubiquitin conjugating enzyme E2 I
UCHL: Ubiquitin C-terminal hydrolases
UPS: Ubiquitin proteosome system
VEGF: Vascular endothelial growth factor
WT1: Wilms tumor 1
WNT4: Wnt family member 4
YAP: Yes-associated protein
ZGLP1: Zinc finger GATA-like protein 1
